# Variance in intraindividual stability of vitreous humor for thanatochemistry

**DOI:** 10.1007/s00414-025-03482-6

**Published:** 2025-04-08

**Authors:** Antonia Kaehler, Piotr Kuta, Thomas Renné, Jack Garland, Rexson Tse, Stefanie Iwersen-Bergmann, Antonia Fitzek, Benjamin Ondruschka

**Affiliations:** 1 Institute of Legal Medicine, University Medical Center Hamburg-Eppendorf, Hamburg, Germany; 2 Institute of Clinical Chemistry and Laboratory Medicine, University Medical Center Hamburg- Eppendorf, Hamburg, Germany; 3 Queensland Public Health and Scientific Services, Coopers Plains, QLD Australia

**Keywords:** Vitreous humor, Postmortem, Thanatochemistry, Postmortem interval

## Abstract

**Introduction:**

This study investigates the intraindividual variability and ex-vivo stability of vitreous humor (VH) analytes for forensic thanatochemistry. VH, an anatomically isolated and robust postmortem fluid, provides valuable insights into the postmortem interval (PMI) and potential antemortem medical conditions.

**Materials and methods:**

A total of 207 VH samples were collected from 36 cases and divided into three cohorts: serial sampling across three days postmortem, intraindividual variability assessment using triplicates, and ex-vivo stability evaluation through freeze-thaw cycles.

**Results:**

Linear relationships between PMI and potassium, calcium, creatinine, and lactate were identified, but their non-linear trends limited PMI estimation accuracy. Sodium, chloride, and glucose demonstrated variations linked to cardiovascular and infectious conditions, while elevated urea and creatinine correlated with renal or multi-organ failure. The study highlighted the importance of interpreting these biochemical markers in conjunction with clinical and environmental factors to improve reliability.

**Discussion:**

Ex-vivo stability testing revealed relevant variations in VH levels, emphasizing the need for standardized pre-analytical handling. Overall, while VH analytes offer promising forensic applications, their use for PMI estimation and medical diagnoses requires cautious interpretation within a multidisciplinary context.

**Supplementary Information:**

The online version contains supplementary material available at 10.1007/s00414-025-03482-6.

## Introduction

Postmortem biochemistry, also known as thanatochemistry, is a study of biochemical changes in the human body after death. It can provide further information to the conventional three cavity postmortem examination. Applications of thanatochemistry include postmortem interval estimation, identify electrolyte or metabolic imbalance, and cause of death determination [1–4].

Vitreous humor (VH) is a relatively well studied and a common matrix uniquely used for thanatochemistry. Different from blood and other bodily fluid, VH is easily collected, robust to the postmortem changes and has lower risk of contamination [1, 2, 4–6]. VH is extensively studied for postmortem interval estimation both in adults and paediatric populations [1, 2, 4, 6]. These studies commonly analyse multiple individuals in a single time point rather than serially which may limit its accuracy in postmortem interval estimation [2, 6–8]. Furthermore, these studies seldom taken into account on the effects of cause of death [4, 9–12]. Also, as VH can be used for other applications, VH samples taken can be split for different analysis or kept for further analysis if indicated. However, it is unclear whether VH samples that are split and/or stored differ from when the VH is sampled in total and promptly analysed.

The aims of this study are to investigate the *serial* changes of VH analytes in the postmortem interval with exploration of different causes of death, document any intra individual variability of VH analytes, and explore ex-vivo stability of VH analytes. This would assist in better interpretation of VH thanatochemistry.

## Materials and methods

### Case selection

This study was a single centre prospective study in which 36 consecutive suitable adult deceased cases admitted to the Institute of Legal Medicine Hamburg, Germany was ultimately used for the study within one year. The demographic and anthropometric data, and medical history were recorded for each case.

#### Inclusion criteria

1. A postmortem interval (PMI) of < 40 h.
2. A reasonable pre-described clinical cause of death was declared.

#### Exclusion criteria

1. Samples that were visibly contaminated with blood.
2. Cases showing putrefactive changes.
3. Insufficient sample for analysis.
4. Paediatric population (< 18 years old) and cases that were suspicious/homicide due to legal implications.

### Case stratification

**Fig. 1:**
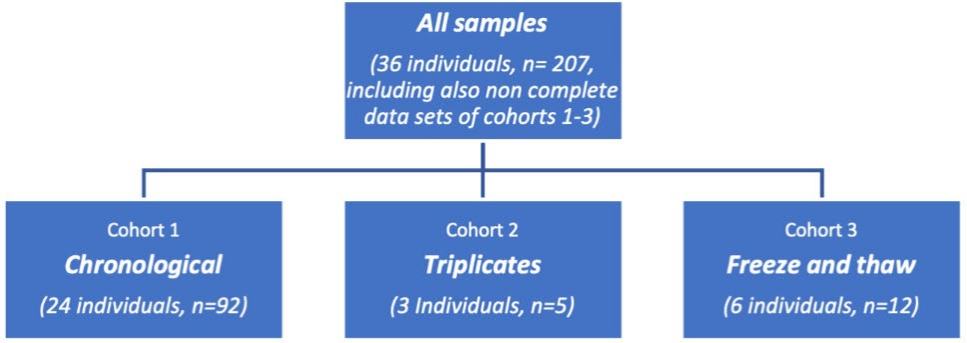
Cohorts, n = number of cases

Of the total 36 cases (207 samples), 33 matched the criteria to be stratified into 3 different groups/cohorts. Three of the measurement series could not be completed due to insufficient samples and/or contamination. Cases and samples from the latter two cohorts described below were different from the first group. Figure 1 illustrates the case stratification.

#### Serial sampling for postmortem changes

To document the changes of VH analytes chronologically, VH was sampled in three different time points being right after death / admission to the morgue (T_0_), and one (T_1_), and two (T_2_) days afterwards (~ 24 h apart). For each time point 1 ml of VH was sampled and analysed. A total of 24 cases and 92 samples were used in this cohort.

#### Intra-individual variability

To explore intra-individual variability, VH was sampled three consecutive times in succession (1 ml each time, to a total of ~ 4 ml) and analysed separately. A total of three cases and five samples were used in this cohort.

#### Ex-vivo stability

To explore the ex-vivo stability, VH was sampled and divided into three aliquots, and subjected to 1 to 3 cycles of freeze thaw cycle. Thawing was done at room temperature until the samples were completely melted. Freezing and refreezing was performed by placing the sample in a calibrated − 20 °C freezer for at > 24 h. A total of six cases and 12 samples were used in this cohort.

### Sampling and handling of VH

VH samples were collected using a standardized approach. For each case, the eyes were punctured through the conjunctiva lateral to the sclero-corneal junction using a sterile 0.8 mm / 40 mm or 21G x 1 1/2 needles (B. Braun Melsungen AG, Melsungen; Germany) and 2 ml or 10 ml syringes (BD Discardit II, Heidelberg; Germany). The VH was aspirated gently to avoid retinal injuries and potential cellular contamination. The eyes were finally re-filled with saline after completion of sampling.

For each sample, the VH was aliquoted in sterile Eppendorf tubes and frozen at a calibrated − 20 °C freezer until analysis. Before measurement, the VH samples were thawed to room temperature, centrifuged at 2000 rpm for 2 min, the supernatant was diluted 1:2 with deionized water and homogenized for 10 s.

### Analysis of VH

For each VH sample, calcium, chloride, sodium, glucose, urea, creatinine and C-reactive protein (CRP) levels were determined using Siemens Attelica Solution analyzers (SiemensHealthineers, Forchheim, Germany) with Siemens reagents in an accredited hospital chemistry laboratory. The hemolysis, icterus and lipemia indices were also determined for each sample for quality control. Limits of quantification (LOQs) were as follows: CRP: 8 mg/L, creatinine: 0.3 mg/dL, chloride: 100 mmol/L, calcium: 0.5 mmol/L, sodium: 100 mmol/L, potassium: 4 mmol/L, glucose: 40 mg/dL, and urea: 10 mg/dL. For potassium, the maximum value was 20 mmol/L. If the measured values were below or above these limits, the value was equated with the given threshold.

### Statistical analysis

Microsoft Excel (version 16.73 for macOS; Microsoft Corporation, Redmond, USA) and Prism (version 10.1.1 for macOS; GraphPad Software Inc., La Jolla, USA) were used for statistical analysis, a p value of < 0.05 was considered statistically significant. For summary statistics, continuous variables were presented as mean, median, standard deviation, minimum and maximum, and categorical variables were presented as counts. Spearman´s rank correlation coefficient was calculated to explore association between continuous variables. For comparative statistics Friedman test followed by post-hoc Dunn´s multiple comparison tests were used. Linear regression analysis was used for predictive statistics.

## Results

### Overall VH sample quality and PMI association

The hemolysis index ranging from 0 (min) to 3 (max) was 0 in the majority of samples (*n* = 207, median = 0, interquartile range = 0). This suggests that the quality of the body fluid was largely unaffected by blood contamination. Over time, there was a statistically significant increase in the hemolysis index (*p* = 0.0009), with a positive linear regression observed (*p* = 0.0136). The hemolysis index showed a significant positive correlation with CRP, calcium, potassium, and lactate levels. In contrast, both the icterus and lipemia indices consistently measured 0 and showed no correlation with the analyzed variables.

### Serial sampling for postmortem changes

#### Population

This group consisted of 12 male and 12 female individuals, with a median age of 74 years (range: 44–99 years). The median body mass index (BMI) was 24.7 kg/m² (range: 16.1–36.4 kg/m²). The documented causes of death were often multifactorial, with 58.3% (*n* = 14) of patients having respiratory conditions; no cases of non-natural causes were reported. Additionally, 45.8% (*n* = 11) had a neoplastic disease, 33.3% (*n* = 8) suffered from an infection, 20.8% (*n* = 5) had a central nervous system illness, 16.7% (*n* = 4) were diagnosed with multiple organ or renal failure, and 12.5% (*n* = 3) had cardiovascular diseases. The median time from death to cooling was 8 h (range: 3.9–22.8 h).

#### Timing of sampling

The median time interval key events were as follows: from death to T0 were 19.8 h (6.1–37.9 h), from T0 to T1, 22.2 h (13.8–42.8 h) and from T1 to T2, 70.8 h (49.3–70.8 h). Detailed descriptive statistics of these measurements are provided in Table 1.

#### CRP

No significant correlation between CRP levels and PMI and no significant linear regression was found. CRP levels in VH did not correlate with different causes of death. There were some intraindividual differences between the left or right eyes of the patients and elevations were only shown in one eye of the individual while the values in the other were beneath the measurement range. Additionally, elevated CRP levels were typically observed at only one time point, except in two cases. These two individuals, who had elevated levels at both T1 and T2, died of pneumonia and sepsis.

#### Sodium

**Fig. 2:**
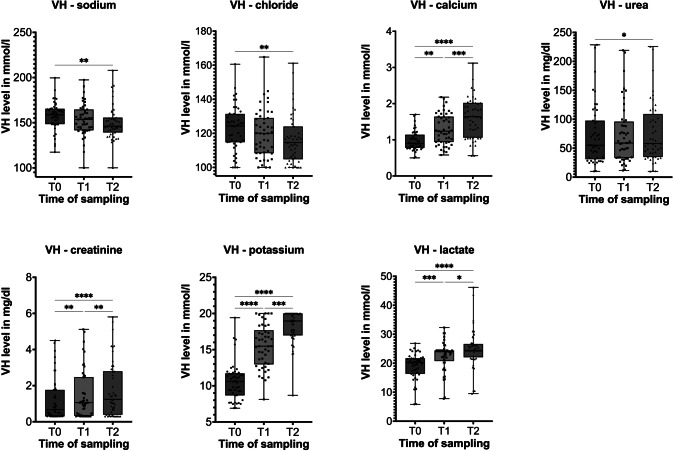
Time dependency of various VH analytes. Dot and box plot diagrams of seven different laboratory parameter levels at the three different postmortem sampling times. *-**** = grade of significance * = *p* ≤ 0.05, ** = *p* ≤ 0.01, *** = *p* ≤ 0.001, **** = *p* ≤ 0.0001. VH, vitreous humor. Results for CRP and glucose has not been plotted as most of the measurements were below the limit of quantification. The results are described in the main manuscript

The median levels of sodium concentration decreased from T_0_ to T_1_ and T_2_ with a significant decrease between T_0_ and T_2_ but linear regression was not significant (*p* = 0.4898), see Fig. 2 + 3.

There were significant relations of sodium levels to infectious as well as cardiovascular causes of death.

#### Chloride

A chronological decrease in chloride VH level was observed, with statistical significance documented between T_0_ and T_2_, however, linear regression was again not reaching significance (*p* = 0.06), see Fig. 2 + 3.

Significant correlations could be found between cardiovascular and infectious diseases and chloride levels.

#### Calcium

Calcium VH levels showed an increase over time. This pattern was found to be strongly significant in all the three examined intervals. Linear regression was also significant (*p* < 0.0001) with the equation PMI = 24.67*[Ca] + 13.55, see Fig. 2 + 3.

Calcium levels correlated with cardiovascular causes of death.

#### Glucose

There was no significant variation observed between the different measurement times of glucose levels in VH, which was confirmed by a non-significant linear regression (*p* = 0.48).

Cardiovascular, respiratory, neoplastic, central nervous and infectious causes of death showed a significant relation to VH glucose. Three individuals showed elevated levels of glucose in all intraindividual measurements, whereas one died of respiratory insufficiency, one of renal failure caused by exsiccosis and one of cardiac decompensation. The sum of Traub ($\:\left[\text{g}\text{l}\text{u}\text{c}\text{o}\text{s}\text{e}\right]+\left(\right[\text{l}\text{a}\text{c}\text{t}\text{a}\text{t}\text{e}]/2$) varied intraindividual in most probands. There was one case with all intraindividual values stayed below 160 mg/dl and none with elevated levels above 410 mg/dl. The case with low sum of Traub had a diagnosed hypoglycemia as cause of death.

#### Urea

**Fig. 3:**
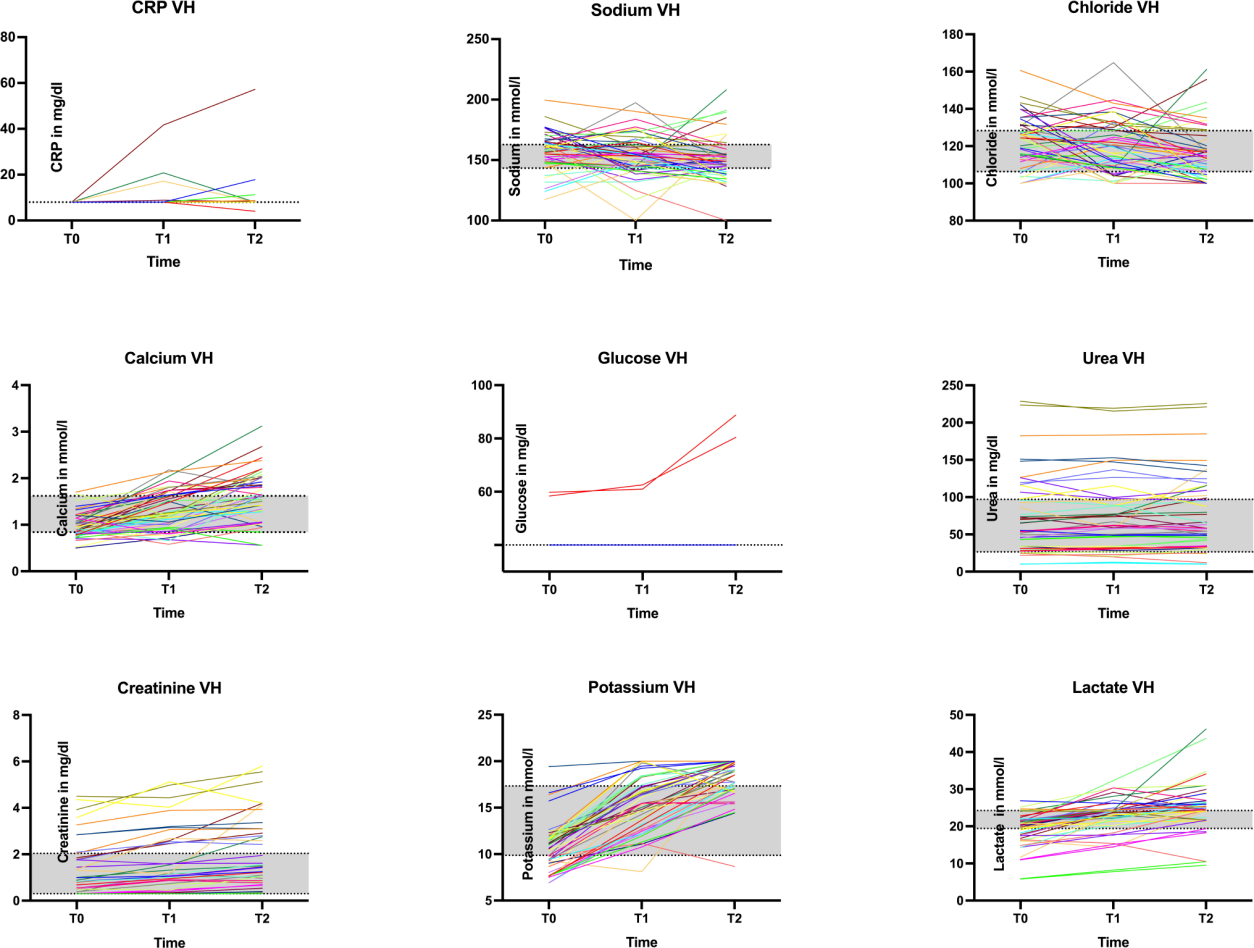
Concentration of the investigated parameters in relation to increasing time since death, dotted lines show the 25th and 75th percentile of all measurements, grey area shows the interquartile range. All individual values or lines outside the grey area were outside the interquartile range of measurement

A significant increase in urea levels was observed from T_0_ to T_2_; however, linear regression analysis did not demonstrate statistical significance (*p* = 0.09), as shown in Figs. 2 and 3. Urea concentrations in vitreous humor (VH) were significantly correlated with renal or infectious causes of death and multi-organ failure.

In four cases, urea levels in VH ranged between 100 and 200 mg/dL. According to the death certificates, these individuals suffered from acute renal failure, metastasized malignancies, and pulmonary edema. In two additional cases, urea levels exceeded 200 mg/dL; one of these individuals was diagnosed with renal failure, while the other had pneumonia.

#### Creatinine

The study showed a consistent and strongly significant increase of creatinine levels in VH within the observed intervals. Consequently, linear regression verified this calculation with the equation $\:\text{P}\text{M}\text{I}\:=\:2.988\text{*}\left[\text{C}\text{r}\text{e}\text{a}\right]\:+\:40.82\:(\text{p}\:=\:0.0186)$, see Fig. 2 + 3.

Creatinine levels were significantly related to renal or infectious cause of death and multi-organ failure.

Elevated creatinine levels above the threshold of 2 mg/dl were found in the same cases where also elevated urea levels were detected. Two other cases showed elevated creatinine levels in only some of the samples, one with also elevated urea, the other without.

Interestingly, creatinine and urea levels correlated positively and highly significant (*r* = 0.856, *p* < 0.0001).

#### Potassium

Potassium levels constantly increased over time, with a strongly statistically significant correlation for all intervals. The linear regression (*p* < 0.0001) resulted in the formula $\:\text{P}\text{M}\text{I}\:=\:4.315\text{*}\left[\text{K}\right]\:\--\:18.54$, see Fig. 2 + 3. Potassium VH levels showed significant correlations with cardiovascular and neoplastic diseases.

Table 2 compares the differences between real and estimated PMI in different intervals using published formulas with our values.

#### Lactate

A significant trend over time was shown as the lactate concentration of vitreous humor increased. A highly significant positive linear regression (*p* < 0.0001) resulted in the equation$\:\:\text{P}\text{M}\text{I}\:=\:1.963\text{*}\left[\text{L}\text{a}\text{c}\right]\:+\:1.339$, see Fig. 2 + 3.

Lactate levels correlated significantly with neoplastic causes of death.

High glucose and high lactate did not correlate significantly (*r* = -0.03, *p* = 0.7).

Table 3 compares the differences between real and estimated PMI in different intervals using published formulas with our values.

### Intra-individual variability

Measuring triplicates showed maximum differences between − 8.7% to + 6.6% in potassium (maximum coefficient of variation 5.1%), -9.0% to + 9.2% in lactate (CV 5.9%) and up to 5.0% in creatinine (CV 2.6%). Urea levels showed deviations of -12.8% to + 10.1% (CV 7.2%) and glucose levels of -2.4% to + 10.1% (CV 6.5%). Deviations of -12.7% to + 5% were evident in calcium (CV 7.1%), and − 4.6% to + 11% in chloride (CV 6.8%) as well as -4.9% to + 12% in sodium (CV 6.1%). CRP levels stayed constantly below the LOQ in all VH sampled for this cohort. Repeated measurement of the same sample did not show any deviations in hemolysis indices (*n* = 5).

### Ex-vivo stability

Repeated freezing and thawing caused variations in analyte levels as follows: lactate ranged from − 16% to + 10% (maximum coefficient of variation [CV]: 9.2%), potassium from − 29% to + 25% (CV: 17.9%), and creatinine from − 52% to + 16% (CV: 35.1%). Urea levels fluctuated between − 25% and + 12% (CV: 15.1%), glucose up to 8% (CV: 3.9%), and calcium from − 26% to + 40% (CV: 19.4%). Chloride levels varied between − 17% and + 31% (CV: 13.3%), while sodium ranged from − 23% to + 24% (CV: 14%) relative to their initial values. CRP levels did not exceeded levels of quantification in this cohort. Importantly, freezing and thawing did not alter the hemolysis index (*n* = 12).

**Table 1:** Descriptive statistics, iqr = interquartile range, mr = measurement range, loq = limit of quantification

	CRP (mg/dl)	Sodium (mmol/l)	Chloride (mmol/l)	Calcium (mmol/l	Glucose (mg/dl)	Urea (mg/dl)	Creatinine (mg/dl)	Potassium (mmol/l)	Lactate (mmol/l)
n	207	207	207	207	207	207	207	206	205
minimum	1 *(LOQ)*	100 *(LOQ)*	100 *(LOQ)*	0.5 *(LOQ)*	40 *(LOQ)*	10 *(LOQ)*	0.3 *(LOQ)*	6	5.8
maximum	57.26	208	164.8	3.12	88.8	228.6	5.8	20 *(LOQ)*	46.2
median	8	152.8	117.4	1.24	40	55.2	0.72	13.7	23.3
mean	8.6	154.1	119.6	1.28	42.12	70	1.29	13.9	22.6
IQR	-	18.8	20.4	0.74	-	70	1.74	6.8	4.5
MR	49.26	108	64.8	2.62	48.8	218.6	5.5	14	40.4

**Table 2:** Different equations using VH potassium to estimate PMI compared with our potassium levels. MD = median deviation, mda = median of amount of deviation

	Sturner and Gantner [13]	James et al. [8]	Jashnani et al. [2]	Mihailovic et al. [14]	Kaehler et al.
equation	PMI=7.14*[K]–39.1	PMI=4.32*[K]–18.35	PMI=1.076 *[K]–2.815	PMI=2.749*[K]–11.978	PMI=4.315*[K]–18.54
max. PMI	100 h	80 h	50 h	30 h	80 h
MD (all)	20.4	1.2	-32.8	-15.4	0.9
MD (T0)	14.6	7.3	-12.8	-3.5	7.1
MD (T1)	25.6	2.1	-32.6	-18.3	1.8
MD (T2)	23.6	-7.9	-52.7	-29.8	-8.2
MDA (all)	20.4	7.8	32.4	15.4	7.7
MDA (T0)	14.7	7.5	12.6	4.6	7.2
MDA (T1)	25.3	5.8	32.4	18.3	5.8
MDA (T2)	23.7	10.6	52.7	29.8	10.7

**Table 3:** Different equations using VH lactate to estimate PMI compared with our lactate levels. MD = median deviation, mda = median of amount of deviation

	Mihailovic et al. [15]	Bertaso et al. [7]	Kaehler et al.
equation	PMI=1.696*[Lac]-10.562	PMI=([Lac]-20.293)/0.1682	PMI (hours) = 1.963*[Lac] + 1.339
PMI	24 h	141 h	80 h
MD (all)	-16.4	-30.7	1.8
MD (T0)	0.2	-25.9	17
MD (T1)	-19.1	-28.3	-1.5
MD (T2)	-37.8	-40.5	-18.9
MDA (all)	16.7	31.1	14
MDA (T0)	6.7	25.9	16.9
MDA (T1)	17.8	28.3	8
MDA (T2)	37.7	45.6	18.6

## Discussion

The objective of this study was to determine the intraindividual postmortem changes in different markers in VH to help assess the PMI, as well as the predictability of antemortem medical conditions from postmortem VH values. Main results underline a connection between elevated creatinine and urea levels with renal or multi organ failure as well as a low sum of Traub as indicator of a fatal hypoglycaemia. Potassium, calcium, creatinine and lactate showed significant linear relationships with PMI. However, no equation was found to determine the time of death without deviations. On the other hand, the levels of urea remained stable, while those of sodium, chloride and glucose varied in or outside the interquartile ranges.

### Postmortem changes

The precise estimation of time between death and the investigation of a corpse, the PMI, is important in legal questions, as it may help to proof alibis of suspects in case of foul play [2, 16]. Many attempts have been made to determine the PMI using thanatochemistry in VH in the last 60 years. The strength of this study compared to others is the repeated intraindividual measurement in different time intervals, as this was only performed by Mihailovic et al., who analysed potassium changes and by Zilg et al. to analyse glucose and lactate [14, 17].

### Potassium

VH potassium is routinely used in forensic practice to estimate the postmortem interval (PMI) [2, 5, 6, 18]. Antemortem, potassium levels in VH are slightly higher than in blood plasma due to active transport across the ciliary body and lens capsule [2, 4, 6]. Postmortem, potassium shows a significant linear increase with PMI, explained by diffusion through cell membranes as active transport ceases, eventually reaching equilibrium between VH and cell plasma [2, 4, 6, 19].

Various equations for PMI estimation have been developed since the 1960s, with differences arising from distinct time intervals studied [2, 6, 8, 14]. Coe identified a biphasic potassium increase, with higher rates in the first 24 h, rendering single linear formulas less reliable over extended intervals, where potassium levels follow an s-shaped curve [17, 20].

The accuracy of PMI estimation using potassium alone is limited due to intraindividual, environmental (e.g., temperature), and analytical factors, as well as antemortem conditions such as electrolyte imbalances, prolonged agony, or elevated alcohol levels [2, 4, 13, 18, 21–25]. Attempts to include additional variables like hypoxanthine, body temperature, or age have been proposed but are beyond this study’s scope [8, 25]. In conclusion, VH potassium is most reliable for early postmortem PMI estimation but has limited standalone accuracy [4].

## Lactate

Lactate concentration correlates significantly with increasing PMI but is not yet widely used for time-of-death estimation [5, 7, 15]. Tested equations showed deviations due to lactate increases caused by hypoxia, glycolysis, and fermentation during agony and postmortem, making it unpredictable without context [4, 26–28]. No reliable equation currently exists for using VH lactate to determine PMI. As ambient temperature influences metabolic and putrefactive processes, future studies should incorporate body core temperature profiles.

### Creatinine

Creatinine increased significantly after death in VH. Mitchell et al. also found significant correlation between creatinine levels and PMI, which they explained by the dehydration of the eyeball according to time [29]. Our equation led to a general median deviation of − 2.3 h and − 2 h in T_1_, but much higher median differences in T_0_ (22.7 h) and T_2_ (-25 h) making creatinine an unreliable parameter to determine time of death.

### Glucose

It is well noted that glucose levels in VH decrease by 35–70% per hour in the first 6 h after death caused by glycolysis and then remain stable for days afterwards [28, 30, 31]. Here, levels were mostly below LoQ, which corresponds to findings of other authors that healthy individuals have VH glucose levels around zero after 6 h [17]. As all but one T0 intervals were longer than 6 h for this study, no predictable deviation had been expected.

#### Association to causes of death

Generally, it is not recommended to diagnose the cause of death solely biochemically, but the measurements may help to find out more about the death circumstances in combination with other factors [4].

#### Sodium and chloride

In this study, VH sodium and chloride levels remained mostly stable, aligning with previous findings. Postmortem VH sodium often matches serum concentrations in short PMIs, but deviations may indicate antemortem electrolyte imbalances [2, 4, 17, 20, 32–35]. Hyponatremia, linked to polydipsia, freshwater drowning, or adrenal failure, was not observed in this cohort [33, 36–38]. Elevated sodium and chloride levels may result from dehydration, saltwater drowning, or high solute intake, though most samples (n 35 of 36 individuals) exceeded the saltwater drowning cutoff, raising questions about its reliability [36, 39–44]. Preanalytical factors, such as hospital infusions, likely influenced results [45]. Conversely, some studies noted postmortem decreases due to diffusion gradients, potentially impacting diagnostic reliability [17, 29, 30, 46, 47].

## Glucose and lactate

Diagnosing death by hyperglycemia or diabetic coma can be challenging, but VH glucose levels may provide clues [17]. Antemortem VH glucose is typically 50–85% of plasma levels [32, 48, 49]. After death, ongoing glucose metabolism converts it to lactate, complicating early postmortem hypoglycemia diagnosis [12, 17]. Fatal hyperglycemia can be identified by VH glucose above 10 mmol/L or Traub’s sum values over 410 mg/dL one day postmortem, though no samples in this study reached these thresholds [30, 31, 50–52].

Traub’s sum formula remains widely used, but postmortem lactate increases risk overestimating glucose metabolism death. Some authors recommend relying solely on glucose levels for diagnosing hyperglycemia [17, 31, 53, 54]. In this study, a single case with Traub values below 160 mg/dL, indicating hypoglycemia, corresponded to a hypoglycemic death [52].

## Urea and creatinine

VH urea and creatinine levels are lower than in blood but correlate positively between postmortem VH and antemortem blood levels in animals [55, 56]. Elevated urea suggests renal dysfunction, gastrointestinal hemorrhage, or electrolyte imbalance, while high creatinine indicates renal malfunction, protein intake, or heat shock [40, 50]. This study found elevated creatinine significantly linked to age, infections, renal, and multi-organ failure, aligning with existing literature. Urea and creatinine levels were typically elevated together, with most cases consistently above or below thresholds (urea: 100 mg/dL, creatinine: 2 mg/dL), supporting VH as a reliable fluid for diagnosing antemortem renal diseases [40, 56].

### Dehydration pattern

According to other authors, postmortem diagnosis of hypertonic dehydration may be supported by an elevation of sodium > 155 mmol/, chloride > 135 mmol/l and urea > 40 mmol/l [29, 43]. In our study, combined levels above these thresholds were found in 12 individuals while the single corpse with consistently increased values over time indeed died of renal failure caused by exsiccosis.

#### Pre-analytical treatment

In literature, different approaches to liquefy the specimens were made [4, 18, 34, 57–59]. For measuring electrolytes and glucose, blood gas instruments were found to be helpful to avoid dilution [60]. For this study, we used a practicable and cost-effective method to examine the VH samples. Samples that were only frozen and thawed to room temperature led to measurement errors caused by the high viscosity. A good workability was found when the supernatant was diluted 1:2 with deionized water and homogenized for 10 s after centrifugation, as previously described [61].

### Triplicates

A coefficient of variation below 10% is used as threshold for acceptable robustness [62, 63] and this condition was fulfilled for all parameters tested here.

#### Freeze and thaw

Freeze-thaw robustness was demonstrated for all parameters except creatinine when changes stayed within 20% of initial values, per Hess et al. [64]. A stricter 10% threshold, however, would challenge most parameters [62, 63]. These findings highlight that repeated freezing and thawing compromises measurement quality and should be avoided.

## Limitations

The VH sampling was conducted at varying PMIs and only once daily due to personnel limitations, as corpses were admitted at different times. After admission, the corpses were cooled at 4 °C, making time of death estimation dependent on this altered ambient temperature. None of the cases underwent autopsy, so the exact cause of death was not determined, only suggested by the death certificate, acknowledging potential discrepancies between clinical and autopsy findings [65, 66].

In future studies, VH data should be assessed in comparison with analytes in the serum and other compartments, such as pericardial fluid, in individual cases and with a sufficient case load as part of a multi-compartment analysis. This would allow for the comparison of marker profiles in different bodily fluids and the establishment of reference intervals for a spectrum of major causes of death. However, both topics were beyond the scope of the present study.

Despite expert sampling, contamination with blood during puncture cannot be entirely ruled out, which led to measuring the hemolysis index to confirm blood-free samples in most cases. Electrolyte concentrations vary within different parts of the eye, potentially biasing intra-sample differences [2, 67]. Additionally, some samples were insufficient for analysis due to limited volume or high viscosity. The lack of international consensus on postmortem VH sample pre-processing further complicates parameter analysis and inter-laboratory comparability [20, 29].

## Conclusion

In conclusion, while linear equations for various parameters in VH, particularly potassium, can assist in estimating PMI, they should not be relied upon alone. Other factors, such as rigor mortis, body core temperature, and supravital reactions, must also be considered. Distinct parameters may provide valuable insights into potential antemortem medical conditions and causes of death. To ensure accurate measurements, it is essential to liquefy samples properly and avoid repeated thawing of stored samples.

## Electronic supplementary material

Below is the link to the electronic supplementary material.

Supplementary Material 1
