# Supplementary material for: Variance in intraindividual stability of vitreous humor for thanatochemistry
Source: Int J Legal Med. 2025 Apr 8;139(5):2165–75. doi: 10.1007/s00414-025-03482-6 (PMC12354637; doi:10.1007/s00414-025-03482-6)
Supplement: Supplementary file 1 — Supplementary Material 1 [file 414_2025_3482_MOESM1_ESM.pdf]

Institut für Rechtsmedizin

HAMBURG

DAkkS Deutsche Akkreditierungsstelle D-PL-13278-02-01

Prof. Dr. med. B. Ondruschka
Direktor des Instituts

Zentrum für Diagnostik

www.uke.de/rechtsmedizin

UKE • Institut für Rechtsmedizin • Butenfeld 34 • 22529 Hamburg

International Journal of Legal Medicine

To the Editor-in-Chief

Ansprechpartner/in:
Prof. Dr. med. B. Ondruschka

Telefon: (040) 7410-52130
Telefax: (040) 7410-59383

b.ondruschka@uke.de

10.03.2025 On

First revision: "Variance in intraindividual stability of vitreous humour for thanatochemistry"

Dear Editor-in-Chief,

I hope this message finds you well.

Please find attached the revised version of our original article on the intraindividual stability of postmortem biochemical analyses of vitreous humour. We have carefully considered and implemented the suggested changes by both reviewers.

We sincerely hope that the manuscript now fully meets the quality standards for acceptance and publication. Should you require any further information to assist in your evaluation, please do not hesitate to contact me.

Thank you for your time and consideration.

Best regards,

Sincerely,

Prof. Dr. med. B. Ondruschka

Institutsdirektion:
Prof. Dr. B. Ondruschka
Direktor
b.ondruschka@uke.de

Ltd. OA PD Dr. A. Heinemann
Stellvertretender Direktor
heinemann@uke.de

Fr. Fahrenkrug, Fr. Melius, Fr. Moos
Sekretariat Institutsdirektion
ifrhh@uke.de

Forensische Pathologie:
OA Prof. Dr. J. Sperhake
jan.sperhake@uke.de

Forensische Bildgebung:
Ltd. OA PD Dr. A. Heinemann
heinemann@uke.de

Klinische Rechtsmedizin:
OÄ Prof. Dr. D. Seifert
d.seifert@uke.de

Lehre / Weiterbildung:
OA Prof. Dr. S. Anders, MME
s.anders@uke.de

Forensische Toxikologie / Forensische Alkohologie:
PD Dr. S. Iwersen-Bergmann
s.iwersen-bergmann@uke.de

Forens. Molekularbiologie:
Dr. C. Augustin
augustin@uke.de

Forens. Anthropologie / Forens. Altersdiagnostik:
Dr. E. Jopp-van Well, M.A.
e.jopp@uke.de

Postmortale Gewebespende / Hornhautbank:
PD Dr. O. Hellwinkel
hellwinkel@uke.de

Rechtsmedizinischer Bereitschaftsdienst
24 h / 7 Tage erreichbar
Telefon: (040) 7410 - 52127
Telefax: (040) 7410 - 59383

DIN EN ISO 9001:2015
Zertifikat Nr. Z13084

Universitätsklinikum Hamburg-Eppendorf
Körperschaft des öffentlichen Rechts
Gerichtsstand: Hamburg
USt-ID-Nr.: DE21 8618 948

Vorstandsmitglieder:
Prof. Dr. Burkhard Göke (Vorsitzender)
Prof. Dr. Blanche Schwappach-Pignataro
Joachim Prölß
Marya Verdel

Bankverbindung:
Hamburger Sparkasse
Kto.-Nr.: 1280 / 170 638
BLZ: 200 505 50
IBAN-Nr.: DE91200505501280170638
BIC: HASPDEHHXXX
